# Differential affinity of mammalian histone H1 somatic subtypes for DNA and chromatin

**DOI:** 10.1186/1741-7007-5-22

**Published:** 2007-05-11

**Authors:** Mary Orrego, Imma Ponte, Alicia Roque, Natascha Buschati, Xavier Mora, Pedro Suau

**Affiliations:** 1 Departamento de Bioquímica y Biología Molecular, Facultad de BiocienciasUniversidad Autónoma de Barcelona, 08193 Bellaterra, Barcelona, Spain; 2 Universidad Autónoma de Manizales. Colombia; 3 Departamento de Matemáticas, Facultad de Ciencias, Universidad Autónoma de Barcelona, 08193 Bellaterra, Barcelona, Spain

## Abstract

**Background:**

Histone H1 is involved in the formation and maintenance of chromatin higher order structure. H1 has multiple isoforms; the subtypes differ in timing of expression, extent of phosphorylation and turnover rate. In vertebrates, the amino acid substitution rates differ among subtypes by almost one order of magnitude, suggesting that each subtype might have acquired a unique function. We have devised a competitive assay to estimate the relative binding affinities of histone H1 mammalian somatic subtypes H1a-e and H1° for long chromatin fragments (30–35 nucleosomes) in physiological salt (0.14 M NaCl) at constant stoichiometry.

**Results:**

The H1 complement of native chromatin was perturbed by adding an additional amount of one of the subtypes. A certain amount of SAR (scaffold-associated region) DNA was present in the mixture to avoid precipitation of chromatin by excess H1. SAR DNA also provided a set of reference relative affinities, which were needed to estimate the relative affinities of the subtypes for chromatin from the distribution of the subtypes between the SAR and the chromatin. The amounts of chromatin, SAR and additional H1 were adjusted so as to keep the stoichiometry of perturbed chromatin similar to that of native chromatin. H1 molecules freely exchanged between the chromatin and SAR binding sites. In conditions of free exchange, H1a was the subtype of lowest affinity, H1b and H1c had intermediate affinities and H1d, H1e and H1° the highest affinities. Subtype affinities for chromatin differed by up to 19-fold. The relative affinities of the subtypes for chromatin were equivalent to those estimated for a SAR DNA fragment and a pUC19 fragment of similar length. Avian H5 had an affinity ~12-fold higher than H1e for both DNA and chromatin.

**Conclusion:**

H1 subtypes freely exchange *in vitro *between chromatin binding sites in physiological salt (0.14 M NaCl). The large differences in relative affinity of the H1 subtypes for chromatin suggest that differential affinity could be functionally relevant and thus contribute to the functional differentiation of the subtypes. The conservation of the relative affinities for SAR and non-SAR DNA, in spite of a strong preference for SAR sequences, indicates that differential affinity alone cannot be responsible for the heterogeneous distribution of some subtypes in cell nuclei.

## Background

Histone H1 is involved in the formation and maintenance of chromatin higher order structures. It is currently accepted that H1 could have a regulatory role in transcription through the modulation of chromatin folding. H1 has been described as a general transcriptional repressor because it contributes to chromatin condensation, which limits the access of the transcriptional machinery to DNA. Other studies indicate that H1 might regulate transcription at more specific level, participating in complexes that either activate or repress specific genes [1-9]. Preferential binding to SARs (scaffold-associated regions) [10] and participation in nucleosome positioning [11] are other mechanisms by which H1 could contribute to transcriptional regulation. H1 has also been implicated in the inhibition of chromatin replication [12-14].

H1 histones from metazoa have a characteristic three-domain structure: a short amino-terminal domain (20–35 amino acids), a central globular domain (~80 amino acids) and a long C-terminal domain (~100 amino acids) [15]. The N- and C-terminal domains are extremely basic. The C-terminal domain is the primary determinant of H1 binding to chromatin [16]. Several properties of linker histones, such as the ability to stabilize chromatin folding [17,18], the preferential binding to SARs [19], activation of apoptotic nuclease [20] and binding to heterochromatin protein HP1α [21], appear to be determined by the C-terminal domain.

H1 has multiple isoforms. The sequences of over 100 subtypes from plants, invertebrates and vertebrates are available. Often more than one subtype is expressed in a given species. In mammals, at least six somatic subtypes, H1a-e and H1°, a male germ-line specific subtype, H1t, and an oocyte-specific subtype, H1oo, are expressed [22-25]. The subtypes differ in their timing of expression [26], extent of phosphorylation [27] and turnover rate [28,29]. *In vitro *evidence supports the hypothesis that the subtypes differ in their ability to condense chromatin [30-33]. In vertebrates, the amino acid substitution rates differ among subtypes by almost one order of magnitude, suggesting that each subtype could have acquired a unique function [34]. Developmental and gene expression studies also support that the different subtypes play distinct roles in chromatin structure [6,35-37].

Linker histone molecules can exchange *in vitro *and *in vivo *between chromatin binding sites [38-41]. We have used this fact to devise a competitive assay to estimate the relative affinities of the subtypes H1a-e and H1° for long chromatin fragments in physiological salt. The results described here also show that although the absolute affinities of H1 subtypes for different DNA sequences can vary widely, the relative affinities are conserved.

## Results

### Relative affinities of the H1 subtypes for DNA

**Figure 1:**
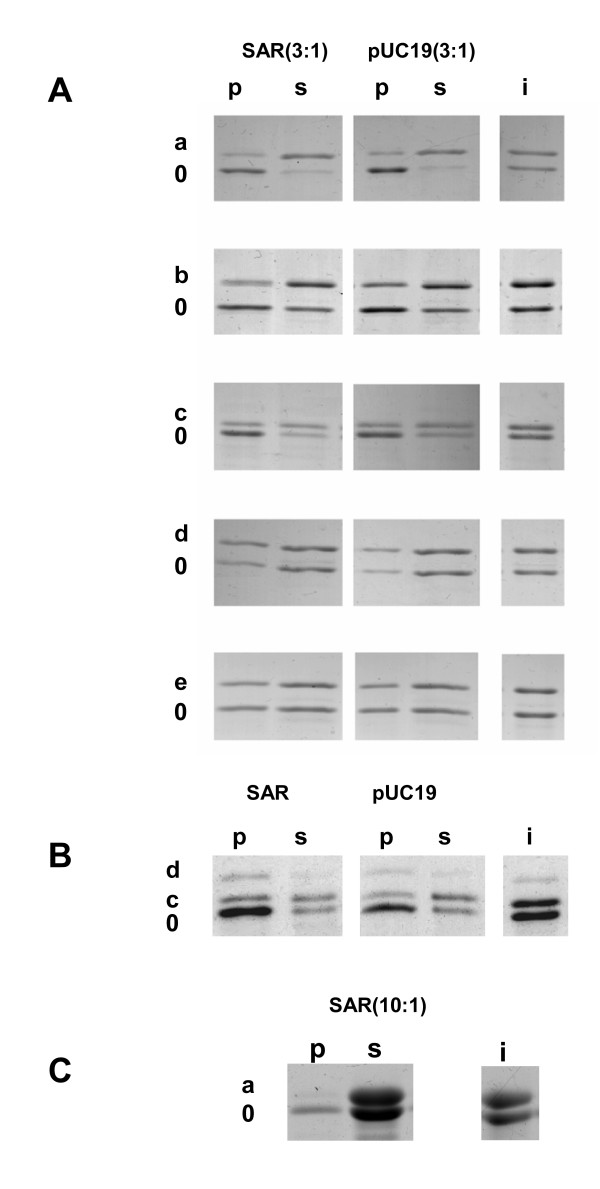
**Competition between H1 subtypes for a limited amount of DNA**. **(A) **Competition between pairs of subtypes. The protein (total H1)/DNA ratio was 3:1 (w/w). This weight ratio is equivalent to ~0.09 H1 molecules per base pair or ~3 H1 molecules per DNA binding site (assuming a DNA binding site of 33 base pairs [42]). The whole pellet and 1/3 of the supernatant were run on the gel. The subtypes H1a-e and H1° are indicated on the left (a-e, 0). SAR, scaffold attachment region from the *Drosophila *histone cluster (657 bp); pUC19, HaeIII/HaeIII fragment from pUC19 (587 bp); i, input mixture of subtypes; p, pellet; s, supernatant. **(B) **Competition between three subtypes. **(C) **Competition with a protein/DNA ratio of 10:1 (w/w), equivalent to ~10 H1 molecules per DNA binding site. The whole pellet and supernatant were run on the gel.

The relative affinities of the H1 subtypes for a SAR fragment of 657 bp from the *Drosophila *histone cluster [10] were estimated by competition of two or three subtypes for a limited amount of DNA (Figure 1). The complexes were prepared by salt-gradient dialysis and their subtype composition did not change with further incubation. Their composition was therefore considered to be at equilibrium or very close to equilibrium. Complexes were separated from the remaining free protein by centrifugation, and the relative affinities of the competing subtypes estimated from the band intensities of the pellets and the supernatants (see Additional File 1) as described in the Methods section with the expression:

*k*_DNA_^i/j ^= ([iDNA] [j]_free_)/([jDNA] [i]_free_)

With increasing excess of a 1:1 mixture of two competing subtypes, the ratio of free subtypes, [j]/[i], tends to one and the ratio of the bands in the pellets approaches the true value of *k*_DNA_^i/j^. Figures 1A and 1C show competition between H1° and H1a with excess protein of 1.5 and 10, which yield values of H1a/H1° in the pellets of about 6 and 15, respectively.

With the six subtypes H1a-e and H1° fifteen different pairs can be formed; however, five pairs, including the six subtypes, are enough to estimate the relative affinities of the six subtypes. We measured the relative affinities of eight (pUC19) or nine (SAR) different pairs (Table 1), and the redundancy of the data was used to calculate the best values fitting the experimental data as well as the error associated with the estimate (Table 2). Representative competitions are shown in Figure 1. The order of affinities for SAR DNA, expressed relative to H1a, the weakest binding subtype, was H1a (1.0)<H1c (4.1)<H1b (5.6)<H1e(15.0)<H1°(20.0)<H1d(20.0), where the figures in parenthesis are the relative affinities.

Similar experiments were performed with a pUC19 fragment of 587 bp. The relative affinities were similar to those obtained with the SAR fragment, namely H1a (1.0)<H1c (5.0)<H1b (6.1)<H1e(15.4)<H1°(20.9)<H1d(24.0). The calculated relative affinities and the absolute error estimates for all possible pairs are given in Tables 2 and 3.

### Relative affinities of the H1 subtypes for chromatin

To estimate the relative affinities of the subtypes for chromatin, we perturbed the H1 complement of native chromatin by adding an additional amount of one of the subtypes. A small amount of SAR DNA was present in the mixture to avoid precipitation of chromatin by excess H1. SAR DNA also provided a set of reference relative affinities, needed to estimate the relative affinities of the subtypes for chromatin from the distribution of the subtypes between the SAR and the chromatin. The amounts of chromatin, SAR and additional H1 were adjusted so as to keep the stoichiometry of perturbed chromatin similar to that of native chromatin. H1 molecules freely exchanged between the chromatin and SAR binding sites, leading to a new chromatin H1 complement enriched in the added subtype. In the presence of 140 mM NaCl, complete equilibration of H1 between chromatin and SAR binding sites was reached in less than 30 min.

We used a SAR sequence in the exchange experiments because the high binding affinity of H1 to SARs allowed the easy separation of the H1/SAR complexes from chromatin after exchange. We estimated that the binding of H1 to the SAR fragment was about 40 times stronger than to the pUC19 fragment (M. Orrego and P. Suau, unpublished results). With polyglutamic acid, a molecular species that has been used as a histone sink in nucleosome and chromatin reconstitution, the insoluble complexes with H1 were contaminated with core histones. The pUC19 fragment used in the binding experiments was also tested but the exchange was very inefficient, probably due to the much higher affinity of H1 for chromatin than for the pUC19.

**Figure 2:**
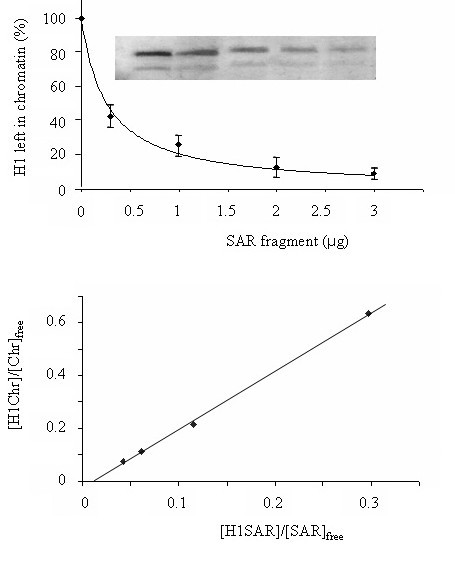
**Displacement of histone H1 from chromatin by SAR DNA**. **(A) **A chromatin sample containing 20 μg of DNA was titrated with a SAR fragment (657 bp). Displaced H1 formed an insoluble complex with the SAR that was separated by centrifugation. The inset shows the H1 remaining on the chromatin. The experimental points were fitted to a hyperbolic function. **(B) **A plot of [H1Chr]/[Chr]_free _on the ordinate and [H1SAR]/[SAR]_free _on the abscissa gives a straight line. The slope of the line corresponds to *k*_Chr_/*k*_SAR_. The points are the average of three experiments. The bars show the interval spanned by the extreme values.

**Figure 3:**
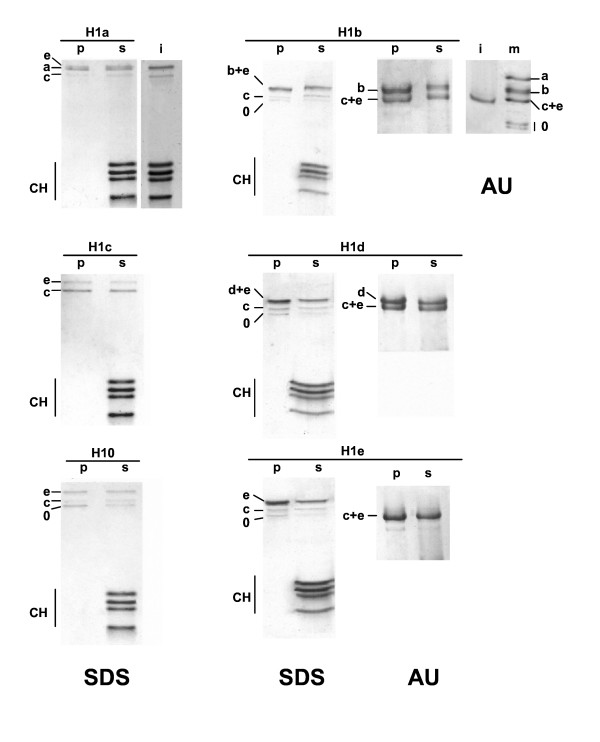
**Perturbation of the H1 complement of native chromatin**. A sample of chromatin (30–35 nucleosomes) containing 30 μg of DNA was mixed with ~8 μg of SAR DNA and ~3 μg of one of the subtypes H1a-e and H1°. This amount of added H1 corresponds to ~0.4 molecules per nucleosome. The insoluble complex of SAR and H1 (p) was separated by centrifugation from chromatin (s) with its H1 complement redefined. Perturbation experiments with H1a, H1c and H1° were analyzed only by SDS gel electrophoresis. Experiments with H1b, H1d and H1e were analyzed by SDS and urea/acetic acid gel electrophoresis. Chromatin inputs analyzed by SDS and AU gel electrophoresis are shown (i). m, mixture of H1 subtypes separated by AU gel electrophoresis. The subtypes H1a-e and H1° are indicated (a-e, 0). CH, core histones.

In the absence of added H1, the SAR fragment efficiently displaced the H1 from chromatin (Figure 2). The affinity of H1 for chromatin binding sites, estimated from the displacement curve as described in the Methods section, was about 2.2 times that for SAR binding sites (assuming SAR and chromatin binding sites of 33 [42] and 198 bp, respectively, and an initial H1 stoichiometry of 0.8 molecules per nucleosome [43]). In this simple displacement experiment it could already be seen that the relative intensities of the H1 bands left on chromatin were similar to those of the original H1 complement, indicating that the relative affinities of the subtypes for chromatin and the SAR were also similar. However, H1a, H1b, H1d and H1° were present at low or very low amounts in chromatin and could not be detected in this kind of displacement experiment. Furthermore, we wished to measure the relative affinities of the subtypes at a stoichiometry similar to that of native chromatin. With this goal in mind, the H1 complement was redefined by adding a certain amount of a purified subtype. Independent experiments were performed with each of the six subtypes (Figure 3). The experimental relative affinities, *k*_Chr_^i/j^, were calculated from the band intensities of the SAR complexes and chromatin (see Additional File 2) as described in the Methods section with the expression:

*k*_crom_^i/j ^= *k*_SAR_^i/j^([iChr]/[iSAR]/[jChr]/[jSAR])

As for the experiments of interaction with DNA, the redundancy of the experimental data allowed the estimate of the best values of relative affinity and the percentage error associated to the estimate.

The experimental relative affinities are shown in Table 4. The values of e/c outnumber all other i/j values because as major subtypes they could be measured in all perturbation experiments. In contrast, H1a, b, d were virtually absent from native chromatin. It is to be noted that the values of e/c were remarkably similar with independence of the added subtype, further indicating that the subtype composition had reached equilibrium. Using these values, the relative affinities best fitting the experimental values were calculated for all possible pairs, together with the absolute error estimates (Table 5). Table 6 shows the overall error estimates resulting from propagation of the errors in the estimates of the relative affinity for the SAR. The average of these estimates expressed in percentage is about 18%.

The relative affinities of the H1 subtypes for chromatin expressed in function of the weakest binding subtype H1a were: H1a (1.0)<H1b (4.2)<H1c (6.4)<H1e (15.9)<H1°(16.3)<H1d(18.9).

The six subtypes can be classified in three groups based on affinity: the high-affinity subtypes H1e, H1d and H1°, the intermediate-affinity subtypes, H1b and H1c, and H1a, the weakest binding subtype. The differences within the groups of intermediate and high affinity are small, and become non-significant when the experimental error is considered, but the entity of the affinity groups is not compromised because the error is small compared to the differences between groups.

### Relative affinity of avian H5 for DNA and chromatin

**Figure 4:**
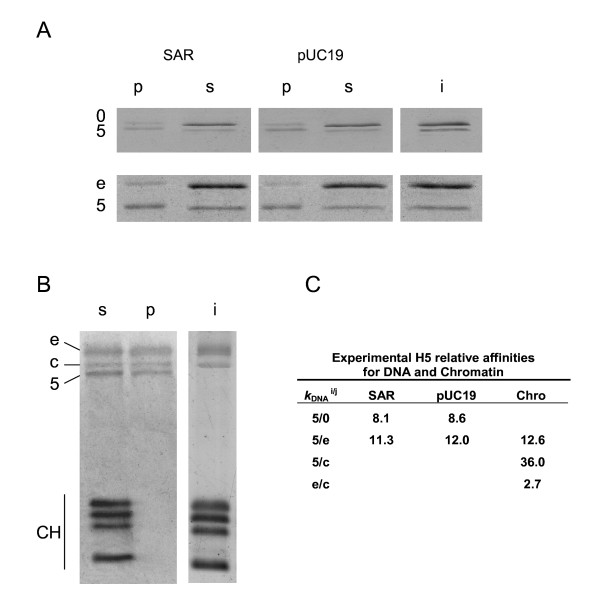
**Affinity of avian H5 for DNA and chromatin**. **(A) **Competition between H5 and H1° or H1e for a limited amount of SAR and pUC19 DNA. **(B) **Perturbation of H1 complement of native chromatin with H5. **(C**) Experimental H5 relative affinities for SAR and pUC19 DNA and chromatin. The number is the average of three independent experiments. p, pellet; s, supernatant; i, input; Chro, chromatin; 0, H1°; 5, H5; e, H1e; c, H1c; CH, core histones.

The relative affinities of chicken H5 and mammalian H1° for the SAR and the pUC19 DNA fragments were obtained from competition experiments as those described for the mammalian subtypes (Figure 4A). H5 had an affinity ~8-fold higher than H1° and ~12-fold higher than H1e for both DNA fragments (Figure 4C).

The native H1 complement of rat liver chromatin was perturbed with H5 to obtain the relative affinity of this heterologous subtype for chromatin. It can be seen that H5 was incorporated into chromatin (Figure 4B). Calculation of H1e/H5 relative affinity with Equation 2 gave a value of ~12 (Figure 4C), which coincides with the relative affinity for SAR DNA. In the same experiments, an H1c/H5 relative affinity of ~36 was obtained. The H1e/H1c relative affinity was 2.7, which is consistent with the previous values shown in Table 4.

## Discussion

We used the free exchange of H1 molecules between chromatin and DNA binding sites to estimate the relative affinities of H1 somatic subtypes H1a-e and H1° for purified chromatin fragments of 30–35 nucleosomes in physiological salt at constant H1 stoichiometry. The assay consists of perturbing the H1 subtype complement of native chromatin by adding a certain amount of each of the purified subtypes. The presence of an appropriate amount of SAR DNA, for which histone H1 has a very high affinity, ensures the maintenance of the original H1 stoichiometry while avoiding chromatin precipitation by excess H1. H1 freely exchanges between chromatin and SAR binding sites. The relative affinities of the subtypes for chromatin can be estimated from the equilibrium distribution of the subtypes between chromatin and SAR, provided the relative affinities of the subtypes for the SAR are known. The latter were obtained from competition experiments between two or three different subtypes for a limited amount of DNA. This method could reproduce some aspects of *in vivo *binding, as, in a situation of free exchange, the occupation of a binding site by a given subtype should be determined by its relative concentration and affinity.

H1a is the subtype with the lowest affinity, H1b and H1c have intermediate affinities and H1°, H1e and H1d have the highest affinities. The lowest and the highest affinities differed by a factor of 19, which corresponds to a ΔG°, of -1.8 Kcal/mol. Such a difference in binding affinity is large enough to support the functional differentiation of the subtypes. The affinities for DNA are similar but not identical to those for chromatin.

To validate the method for the estimation of subtype relative affinities, we performed competition experiments with avian H5. This subtype is restricted to avian nucleated erythrocytes, which are virtually transcriptionally inert. H5 is highly related to H1°, but in contrast to the latter, its C-terminal domain is highly arginine-rich [44]. This feature could contribute to the stability of the chromatin higher-order structure of avian erythrocyte chromatin. H5 appeared to have a binding affinity much higher than any mammalian somatic subtype for both DNA and chromatin: about 12-fold higher than H1e.

The differences in affinity of the H1 subtypes are basically determined by the C-terminal domain of the molecule [16]. The C-terminal domain has little structure in dilute solution, but becomes structured upon interaction with the DNA [45]. This suggests that small differences in the structure of the C-terminus affecting the spatial distribution of basic residues could be important in determining the differential affinity of the subtypes.

The relative affinities might be modulated *in vivo *by phosphorylation and other post-translational modifications such as polyADP-ribosylation, in a cell cycle and cell type specific manner, leading to a large variety of subtype affinities [46-49].

H1 binding is not sequence specific; however, it shows binding preferences for certain DNA sequences. Besides a general preference for A.T-rich regions, H1 preferentially binds and aggregates scaffold-associated regions (SARs) [10]. Preference for particular non-A.T-rich sequences has also been reported [50,51]. The contribution of the features of the DNA sequence, in addition to the properties of the subtypes, to the binding affinity could give rise to an even larger variety of binding strengths.

All mammalian somatic subtypes, H1a-e and H1°, preferentially bind to SARs [19]. Interestingly, the relative affinities of the subtypes for SAR and non-SAR DNA (pUC19) appear to be similar, independently of the overwhelming preference for SAR DNA. The conservation of the relative affinities implies that the binding preferences of the subtypes will not by themselves determine the heterogeneous distribution of some subtypes in cell nuclei, as high and lower affinity chromatin binding sites should be occupied with similar probabilities by the different subtypes. This is consistent with the basically electrostatic character of the interaction of H1 with the DNA; however, the preference of particular subtypes for specific sequences cannot be excluded.

Immunolocalization analysis has shown that H1b,d,e and H1° are heterogeneously distributed in cell nuclei. In contrast, the distribution of H1c is relatively uniform [46,52]. Several observations suggest that the degree of chromatin condensation could differentially affect the affinity of particular subtypes and thus contribute to their heterogeneous distribution. Avian H5 seems to bind to chromatin higher order structures with preference over the H1 subtypes of chicken erythrocytes [53]. Subtypes with preference for heterochromatin have been identified in the dipterans *Chironomus *and *Glyptotendipes *[54,55]. A general effect is provided by core histone acetylation, as it has been shown that the residence time of H1 molecules is shortened in chromatin containing highly acetylated core histones [40], and acetylation is apparently sufficient to decondense chromatin even in the presence of linker histones [56]. The subtypes are also non-randomly distributed with respect to active and inactive chromatin. Actively transcribed sequences are presumably located in less condensed regions of chromatin. Gene expression studies localize H1b in regions of active transcription and H1d and H1e in less active or inactive regions [37,57,58], suggesting the association of the subtypes of high binding affinity with more condensed chromatin and of those of intermediate or low binding affinity with less condensed chromatin.

The affinity of H1 subtypes has been examined by fluorescence recovery after photobleaching in neuroblastoma cells stably expressing N-terminal fusions of GPF to histone H1 subtypes [41]. In spite of the complexities of *in vivo *conditions, in particular, the possible presence of post-translational modifications, these results agree rather well with our *in vitro *estimations for the subtypes H1a,c,d,e and H1°. Only H1b had a higher affinity *in vivo *than *in vitro*. Nuclear localization could have an affect on H1 exchange. It should be noted that in transformed fibroblasts GPF-H1b was localized to heterochromatic regions.

A general conclusion that can be drawn from our results with purified components is that H1 can freely diffuse between chromatin binding sites without the contribution of additional protein factors.

## Conclusion

We have devised a competitive assay to estimate the relative affinities of the histone H1 somatic subtypes H1a-e and H1° for purified chromatin fragments (30–35 nucleosomes) in physiological salt (0.14 M NaCl) at constant H1 stoichiometry. H1a was the subtype with the lowest affinity, H1b and H1c had intermediate affinities and H1d, H1e and H1° the highest affinities. The lowest and the highest affinities differed by a factor of about 19. Such a difference is large enough to suggest that differential affinity could be functionally relevant. The relative affinities of the subtypes for chromatin were equivalent to those estimated for a SAR DNA fragment and a pUC19 fragment of similar length. Avian H5 had a binding affinity ~12-fold higher for DNA and chromatin than H1e. The conservation of the relative affinities of the subtypes for SAR and non-SAR DNA, in spite of a strong preference for SAR sequences, indicates that differential affinity alone cannot be responsible for the heterogeneous distribution of H1 subtypes in cell nuclei.

## Methods

### Preparation of histone H1 subtypes

H1 subtypes were from rat brain [26,27] and H5 from chicken erythrocytes. H1 and H5 were extracted with 0.35 M NaCl by exchange with carboxymethyl Sephadex, following the method of García-Ramírez et al [59]. The mixture of subtypes was digested with alkaline phosphatase to eliminate small amounts of phosphorylated forms that could be present. H1° was purified by gel-filtration chromatography, according to Böhm et al [60]. The subtypes H1a-e were separated by reverse phase HPLC according to Brown et al [35]. The subtypes were obtained as homogeneous peaks, except H1c and H1d, which largely overlapped; however, pure H1c could be recovered at the end of the eluting peak, while H1d was recovered at the beginning of the peak with 1–2% contaminating H1c.

Before being used in binding experiments, all subtypes were subjected to a denaturation/renaturation cycle by stepwise dialysis from 6 M urea into, successively, 3.0, 1.5, 0.7, 0.3 and 0.0 M urea, in 0.2 M NaCl, 0.01 M phosphate buffer, pH 7. Finally, the proteins were dialysed against 0.14 M NaCl, 0.01 M phosphate buffer, pH 7.0. The concentration of protein was estimated by amino acid analysis.

The H1 subtypes were designated with the alphabetic nomenclature of Seyedin and Kistler [61] and Lennox [24]. The equivalent subtypes in the Albig et al [62] numeric system and in the sequence-based Parseghian et al [46,63] system are the following: H1a (H1.1, H1a), H1b (H1.5, H1^S^3), H1c (H1.2, H1^S^1), H1d (H1.3, H1^S^2), H1e (H1.4, H1^S^4).

### Preparation of DNA fragments

A SAR fragment of 657 bp from the histone cluster of *Drosophila melanogaster *was obtained by digestion of p1314 [10] with restriction endonucleases *Kpn*I and *Bam*HI. Another DNA fragment, of 587 bp, was excised from pUC19 by digestion with *Hae*III. Both inserts were separated on agarose gels and electroeluted using a Biotrap chamber (Schleider & Schuell, Dassel, Germany).

### Preparation of chromatin fragments

Nuclei were obtained from livers of adult rats. Livers were homogenized in buffer A (1 M sucrose, 1 mM sodium cacodylate, 25 mM KCl, 0.25 mM spermidine, 0.15 mM spermine, 1 mM EDTA, 1% thiodiglycol, pH 6.5. Nuclei were pelleted by centrifugation through 2 M sucrose at 30000 g for 1 h in buffer A, resuspended in 0.25 M sucrose, 1 mM sodium cacodylate, 25 mM KCl, 0.5 mM spermidine, 0.15 mM spermine, 1% thiodiglycol, 2 mM CaCl_2_, pH 6.5 and digested with micrococcal nuclease (7.5 U/mg DNA for 30 sec at 37°C). The reaction was stopped with 10 mM EDTA and cooling on ice. Chromatin fragments were extracted by overnight dialysis against 10 mM Tris, 1 mM EDTA, 70 mM NaCl, 0.1 mM PMSF, pH 7.4 The chromatin fragments were concentrated up to 8–16 mg/ml (expressed as DNA concentration) with a Centriper YM-10 and separated by centrifugation at 140000 g on a linear (5–20%) sucrose gradient containing 10 mM Tris, 1 mM EDTA, 70 mM NaCl, 0.1 mM PMSF, pH 7.4 Chromatin fragments of 30–35 nucleosomes were selected for the exchange experiments. The histone composition of the chromatin fractions was analyzed by SDS and acetic acid/urea gel electrophoresis [64]. The level of phosphorylation of the H1 subtypes of purified chromatin was negligible, as shown by the absence of lower mobility bands in urea/acetic acid gel electrophoresis of input chromatin (Figure 3).

#### Binding assaysCompetition of H1 subtypes for a limited amount of DNA

The relative affinities of the H1 subtypes for the SAR and the pUC19 were estimated in competition experiments of pairs of subtypes for a limited amount of one of the DNA fragments. A total amount of 1.5 μg of H1 was mixed with 0.5 μg of one of the DNA fragments in a final volume of 90 μl. This weight ratio is equivalent to ~0.09 H1 molecules per base pair, or ~3 molecules per DNA binding site (assuming a binding site of 33 base pairs [42]). In general, an approximately 1:1 mixture of the two competing subtypes was used. Experiments where the two subtypes were imbalanced by up to a factor of twelve or with three competing subtypes were also performed. The complexes were prepared by gradient dialysis from 1 M NaCl down to 0.14 M NaCl in 0.01 M phosphate buffer, pH 7.0, 5% glycerol. The complexes were recovered by centrifugation at 14000 g for 10 min. No DNA was left in the supernatants after centrifugation, as shown by ethidium bromide and silver staining. The subtype composition of the complexes and of the free protein left in the supernatants was analysed by PAGE-SDS after staining with Amido Black.

Protein bands were scanned using Model GS-700 Imaging Densitometer (Biorad) and quantified with the program Multyanalyst v.1 (Biorad). DNA was quantified with Gel Doc 1000 (Biorad) gel documentation system and the program Molecular Analyst (Biorad).

#### Analysis of the binding of H1 subtypes to DNA

Considering the binding equilibriums i+DNA ⇄ iDNA and j+DNA ⇄ jDNA, the relative affinity of the i and j subtypes can be expressed as:

*k*_DNA_^i/j ^= *k*_DNA_^i^/*k*_DNA_^j ^= ([iDNA]/[i]_free_[DNA])/([jDNA]/[j]_free_[DNA])

In the mixture, the relative affinity does not depend on the concentration of free DNA because it is the same for both equilibriums; the relative affinity therefore becomes:

*k*_DNA_^i/j ^= ([iDNA][j]_free_)/([jDNA][i]_free_)

Where the concentration of complexes, [iDNA] and [jDNA], is obtained from the band intensities in the pellets and the concentration of free protein, [i]_free _and [j]_free_, from the band intensities of the supernatants.

#### Displacement of H1 from chromatin by SAR DNA

A sample of chromatin containing 20 μg of DNA was incubated in physiological salt for 90 min at 37°C with increasing amounts of a SAR fragment from the *Drosophila *histone cluster. SAR DNA efficiently displaced H1 from chromatin. The complex of H1 with SAR DNA was separated by centrifugation and chromatin bound H1 remaining in the supernatant analyzed by SDS gel electrophoresis. The relative affinity of H1 for chromatin and SAR DNA was estimated with the expression [65]:

[H1Chr]/[Chr]_free _= *k*_Chr_/*k*_SAR_[H1SAR]/[SAR]_free_

The concentrations of bound and free chromatin and SAR were calculated from the displacement curve using 198 bp (the nucleosome repeat length) and 33 bp [60] as H1 binding sites in chromatin and SAR DNA, respectively, in order to obtain the molarities of binding sites. The H1 stoichiometry was taken as 0.8 molecules per nucleosome [43]. It was also assumed that [bound H1]>> [H1]_free _and, therefore, that all H1 displaced from chromatin was bound to SAR DNA.

#### Perturbation of the H1 complement of chromatin

The H1 complement of native chromatin was perturbed by adding an additional amount of a purified H1 subtype. An appropriate amount of the SAR fragment from the *Drosophila *histone cluster was added to keep H1 stoichiometry approximately constant and as a means of having a set of reference relative affinities. The relative affinities of the H1 subtypes for chromatin were obtained from the distribution of the subtypes between the SAR and the chromatin. The reaction mixture typically consisted in an amount of chromatin containing 30 μg of DNA, ~8 μg of SAR and ~3 μg of one of the purified subtypes. The latter amount corresponds to ~0.4 H1 molecules per nucleosome. The mixture was incubated during 90 min at 37°C in 140 mM NaCl to allow for the exchange of H1 between the chromatin and the SAR binding sites. The exchange reaction was kinetically blocked by passing it through a spun-down Sephacryl gel filtration column equilibrated with 10 mM Tris, 1 mM EDTA, pH 7.4. Without salt, no exchange between the chromatin and SAR binding sites was observed, as shown previously for the exchange of H1 between chromatin fragments [38]. The H1-SAR complexes were separated from soluble chromatin by centrifugation at 14 000 g for 10 min. The histone composition of the chromatin and the SAR complex were analyzed by SDS and acetic acid/urea gel electrophoresis. Perturbation experiments with H1a, H1c and H1° were analyzed only by SDS gel electrophoresis. Experiments with H1b, H1d and H1e had to be analyzed by a combination of SDS and urea/acetic acid gel electrophoresis to estimate the contribution of each subtype [64].

#### Binding of H1 subtypes to chromatin in the presence of additional H1 and a SAR fragment

For a given subtype, i, i+SAR ⇄ iSAR, and i+Chr ⇄ iChr.

The equilibrium expressions are:

*k*_SAR_^i ^= [iSAR]/[i]_free_[SAR]

*k*_Chr_^i ^= [iChr]/[i]_free_[Chr]

The relative affinity constant *k*_Chr_^i^/*k*_SAR_^i ^does not depend on the concentration of free protein, [i]_free_, because it is the same for both equilibriums; therefore:

*k*_Chr_^i^/*k*_SAR_^i ^= [iChr] [SAR]_free_/[iSAR][Chr]_free_

Resolving for *k*_Chr_^i^:

*k*_Chr_^i ^= *k*_SAR_^i^[iChr] [SAR]_free_/[iSAR] [Chr]_free_

The ratio [SAR]_free_/[Chr]_free _is the same for all the equilibrium expressions of the different subtypes present in a given assay; therefore, it cancels out when *k*_Chr _^i ^is expressed as relative affinity constant with respect to another subtype, *k*_DNA_^i/j^:

*k*_Chr_^i/j ^= *k*_Chr_^i^/*k*_Chr_^j ^= *k*_SAR_^i^([iChr]/[iSAR])/*k*_SAR_^j^([jChr]/[jSAR])

or

*k*_Chr_^i/j ^= *k*_SAR_^i/j^([iChr]/[iSAR])/([jChr]/[jSAR])

Where the *k*_SAR_^i/j ^values have been estimated previously and the [iChr], [jChr], [iSAR] and [jSAR] have been obtained from the intensities of the H1 subtype bands separated by electrophoresis. Six different exchange experiments were performed, adding each time a different subtype. Nine different i/j experimental relative affinities were obtained out of the fifteen possible (i/j being equal to j/i).

#### Estimate of the best values fitting the experimental data

The redundancy of the experimental data allowed the calculation of the best values for relative affinity. With six different subtypes only five determinations of relative affinity are necessary, provided they include all six subtypes. Of the fifteen possible pairs of subtypes, we examined nine pairs for their relative affinity for the SAR, eight for their relative affinity for the pUC19 and eight for their relative affinity for chromatin. The best values for the apparent relative affinity constants were calculated from the observed values with the application for multiple regression of the EXCEL program. The estimated values were, as usual, those that minimize the average quadratic error. The experimental values of relative affinity had to be logarithmically transformed in order to have a system of linear equations; this meant that once the logarithmic transformation was reversed the upper limit of the errors was slightly different from the lower limit. Wherever indicated, an average of the two error values is given.

## Authors' contributions

MO carried out a large part of the experimental work described in this paper. NB prepared the chromatin fragments. AR contributed to the experiments with H5. XM performed the statistical analysis. IP assisted with some competition experiments and helped to draft the manuscript. The project was conceived and jointly supervised by IP and PS.

## Supplementary Material

Additional file 1 **DNA data**. Band intensities of pellets and supernatants from competition experiments between H1 subtypes for a limited amount of DNA in Microsoft Word format. Click here for file

Additional file 2 **Chromatin data**. Subtype intensities from H1 complement perturbation experiments in Microsoft Word format. Click here for file

## Figures and Tables

**Table 1:** Experimental values of relative affinity of the H1 subtypes for SAR and pUC19 DNA. The numbers correspond to individual experiments.

***k*_DNA_^i,j^**		**SAR**			**pUC19**	
a/0	0.042	0.056	0.043	0.041	0.051	0.056
b/0	0.294	0.280	0.293	0.291	0.283	0.308
c/0	0.202			0.220		
d/0	1.007	1.118		1.250	1.153	
e/0	0.713	0.801	0.708	0.703	0.681	0.781
e/c	3.776			3.301		
c/a	3.780			5.367		
d/c	4.726			4.409		
b/a	5.040					

**Table 2:** Estimated relative affinities of the H1 subtypes for SAR DNA. The absolute error estimates are shown in parenthesis.

***k*_SAR_^i/j^**	**b**	**c**	**d**	**e**	**0**	**(i)**
a	5.6 (0.7)	4.1 (0.4)	20.0 (3.2)	15.0 (2.1)	20.0 (2.0)	
b		0.7 (0.1)	3.7 (0.6)	2.7 (0.4)	3.6 (0.4)	
c			5.1 (0.7)	3.7 (0.5)	5.0 (0.6)	
d				0.7 (0.1)	1.0 (0.1)	
e					1.3 (0.1)	
(j)						

**Table 3:** Estimated relative affinities of the H1 subtypes for pUC19 DNA. The absolute error estimates are shown in parenthesis.

***k*_pUC19_^i/j^**	**b**	**c**	**d**	**e**	**0**	**(i)**
a	6.1 (1.0)	5.0 (0.6)	24.0 (3.8)	15.4 (2.3)	20.9 (2.3)	
b		0.8 (0.1)	3.9 (0.7)	2.5 (0.4)	3.4 (0.3)	
c			4.8 (0.7)	3.1 (0.4)	4.2 (0.4)	
d				0.7 (0.1)	0.9 (0.1)	
e					1.3 (0.2)	
(j)						

**Table 4:** Experimental values of relative affinity of the histone H1 subtypes H1a-e and H1° for chromatin.

**a/c**	**a/e**	**b/c**	**b/e**	**d/c**	**d/e**	**e/c**	**0/c**	**0/e**
0.162	0.061	0.644	0.269	3.038	1.179	2.661	2.627	1.079
		0.647	0.279	3.015	1.165	2.393	2.671	1.073
						2.322	2.430	0.936
						2.494		
						2.434		
						2.578		
						2.588		
						2.385		
						2.464		
						2.489		
						2.597		

**Table 5:** Estimated relative affinities of the H1 for chromatin. The absolute error estimates of the chromatin perturbation experiments are shown in parenthesis.

***k*_Chr_^i/j^**	**b**	**c**	**d**	**E**	**0**	**(i)**
a	4.2 (0.4)	6.4 (0.3)	18.9 (1.5)	15.9 (1.0)	16.3 (1.2)	
b		1.5 (0.1)	4.5 (0.3)	3.7 (0.2)	3.9 (0.2)	
c			3.0 (0.1)	2.5 (0.1)	2.6 (0.1)	
d				0.8 (0.04)	0.9 (0.05)	
e					1.0 (0.04)	
(j)						

**Table 6:** Overall absolute error estimates resulting from the propagation of the error of the estimates of relative affinity for the SAR.

	**b**	**c**	**d**	**e**	**0**	**(i)**
a	1.1	0.7	4.7	3.1	3.2	
b		0.2	0.9	0.6	0.6	
c			0.8	0.6	0.7	
d				0.14	0.15	
e					0.14	
(j)						
