# Supplementary material for: Differential affinity of mammalian histone H1 somatic subtypes for DNA and chromatin
Source: BMC Biol. 2007 May 11;5:22. doi: 10.1186/1741-7007-5-22 (PMC1890542; doi:10.1186/1741-7007-5-22)
Supplement: Additional file 1 — DNA data. Band intensities of pellets and supernatants from competition experiments between H1 subtypes for a limited amount of DNA in Microsoft Word format. [file 1741-7007-5-22-S1.doc]

Table 1. **Band intensities of pellets and supernatants from competition experiments between H1 subtypes for a limited amount of DNA.**

SAR, scaffold attachment region from the *Drosophila* histone cluster (657 bp); pUC19, HaeIII/HaeIII fragment from pUC19 (587 bp); p, pellet, s, supernatant.

|  |  |  | SAR |  |  |  |  |  | pUC19 |  |  |  |
| --- | --- | --- | --- | --- | --- | --- | --- | --- | --- | --- | --- | --- |
|  | p | s | p | s | p | s | p | s | p | s | p | s |
| H1º | 0.93 | 0.19 | 1,23 | 0,23 | 0,71 | 0,44 | 1,28 | 0,15 | 1,44 | 0,21 | 1,27 | 0,77 |
| H1a | 0.21 | 0.77 | 0,25 | 1,11 | 0,06 | 0,86 | 0,24 | 0,68 | 0,31 | 0,89 | 0,10 | 1,09 |
| H1º | 1.12 | 0,74 | 1.24 | 0,83 | 0,29 | 1,26 | 1.35 | 0,72 | 1.24 | 0,66 | 0,58 | 1,52 |
| H1b | 0,53 | 1.19 | 0,56 | 1.34 | 0,08 | 1,19 | 0,65 | 1.19 | 0,58 | 1.09 | 0,16 | 1,38 |
| H1º | 0,99 | 0,19 |  |  |  |  | 0,97 | 0,28 |  |  |  |  |
| H1c | 0,39 | 0,37 |  |  |  |  | 0,35 | 0,46 |  |  |  |  |
| H1º | 0,43 | 0,97 | 0.39 | 0.87 |  |  | 0,25 | 1.12 | 0.37 | 0.87 |  |  |
| H1d | 0,57 | 1.15 | 0.51 | 1.13 |  |  | 0.36 | 1.29 | 0.50 | 1.02 |  |  |
| H1º | 1.01 | 1.43 | 0,38 | 1,595 | 0,38 | 2,884 | 0.97 | 1.59 | 0,54 | 1,24 | 0.342 | 1.83 |
| H1e | 0.74 | 1.47 | 0,24 | 1,429 | 0,16 | 1,51 | 0.69 | 1.61 | 0,55 | 1,61 | 0.160 | 1.25 |
| H1c | 0.22 | 0.67 |  |  |  |  | 0.47 | 1.03 |  |  |  |  |
| H1e | 0.62 | 0.50 |  |  |  |  | 1.22 | 0.81 |  |  |  |  |
| H1a | 0.34 | 1.35 |  |  |  |  | 0.23 | 1.30 |  |  |  |  |
| H1c | 0.99 | 1.04 |  |  |  |  | 0.94 | 0.99 |  |  |  |  |
| H1c | 0.28 | 0.78 |  |  |  |  | 1.48 | 0.58 |  |  |  |  |
| H1d | 0.95 | 0.56 |  |  |  |  | 3.15 | 0.28 |  |  |  |  |
| H1a | 0.40 | 0.14 |  |  |  |  |  |  |  |  |  |  |
| H1b | 0.72 | 0.05 |  |  |  |  |  |  |  |  |  |  |
| H1º | 0,02 | 0,13 | 0,13 | 0,51 | 0,02 | 0,11 | 0,02 | 0,12 | 0,05 | 0,39 | 0,03 | 0,19 |
| H5 | 0,04 | 0,03 | 0,17 | 0,08 | 0,04 | 0,03 | 0,03 | 0,02 | 0,12 | 0,11 | 0,04 | 0,03 |
| H1e | 0,04 | 0,58 | 0,10 | 1,82 | 0,03 | 0,46 | 0,03 | 0,51 | 0,03 | 0,39 | 0,18 | 0,87 |
| H5 | 0,13 | 0,18 | 0,14 | 0,23 | 0,10 | 0,14 | 0,05 | 0,07 | 0,10 | 0,11 | 0,20 | 0,08 |
|  |  |  |  |  |  |  |  |  |  |  |  |  |
