# Supplementary material for: Differential affinity of mammalian histone H1 somatic subtypes for DNA and chromatin
Source: BMC Biol. 2007 May 11;5:22. doi: 10.1186/1741-7007-5-22 (PMC1890542; doi:10.1186/1741-7007-5-22)
Supplement: Additional file 2 — Chromatin data. Subtype intensities from H1 complement perturbation experiments in Microsoft Word format. [file 1741-7007-5-22-S2.doc]

Table 2. **Subtype intensities from H1 complement perturbation experiments.**

The intensities correspond to equilibrium values of the SAR complexes and chromatin after perturbation with and added subtype. SAR, intensities in the complexes with SAR; Chro, intensities in chromatin. The intensity values of the experiments with H1b, H1d and H1e derive from the combination of SDS and urea/acetic acid gel electrophoresis.

| added subtype | intensities | SAR | Chro | SAR | Chro |
| --- | --- | --- | --- | --- | --- |
|  | H1e | 0,150 | 0,27 |  |  |
| H1a | H1c | 0,07 | 0,17 |  |  |
|  | H1a | 0,23 | 0,37 |  |  |
|  | H1e | 0,32 | 0,42 | 0,44 | 0,18 |
| H1b | H1c | 0,09 | 0,17 | 0,12 | 0,08 |
|  | H1b | 0,68 | 0,64 | 0,95 | 0,29 |
|  | H1e | 0,12 | 0,08 |  |  |
| H1c | H1c | 0,24 | 0,22 |  |  |
|  | H1e | 0,37 | 0,21 | 0,82 | 0,66 |
| H1d | H1c | 0,17 | 0,13 | 0,34 | 0,38 |
|  | H1d | 0,74 | 0,37 | 1,71 | 1,20 |
|  | H1e | 1,12 | 0,73 | 2,21 | 1,29 |
| H1e | H1c | 0,09 | 0,09 | 0,15 | 0,13 |
|  | H1e | 0,17 | 0,21 |  |  |
| H1º | H1c | 0,07 | 0,13 |  |  |
|  | H1º | 0,16 | 0,16 |  |  |
|  | H1e | 0,303 | 1,19 |  |  |
| H1º | H1c | 0,101 | 0,583 |  |  |
|  | H1º | 0,16 | 0,51 |  |  |
|  | H1e | 0,24 | 0,70 |  |  |
| H1º | H1c | 0,09 | 0,36 |  |  |
|  | H1º | 0,16 | 0,34 |  |  |
|  | H1e | 0,27 | 0,31 |  |  |
| H5 | H1c | 0,07 | 0,11 |  |  |
|  | H5 | 0,17 | 0,23 |  |  |
|  | H1e | 0,18 | 0,22 |  |  |
| H5 | H1c | 0,05 | 0,08 |  |  |
|  | H5 | 0,12 | 0,18 |  |  |
|  | H1e | 0,09 | 0,13 |  |  |
| H5 | H1c | 0,02 | 0,04 |  |  |
|  | H5 | 0,08 | 0,14 |  |  |
